# Adult neuroplasticity employs developmental mechanisms

**DOI:** 10.3389/fnsys.2022.1086680

**Published:** 2023-01-24

**Authors:** Todd M. Mowery, Preston E. Garraghty

**Affiliations:** ^1^Department of Otolaryngology, Robert Wood Johnson Medical School, Rutgers, The State University of New Jersey, Piscataway, NJ, United States; ^2^Department of Psychological and Brain Sciences, Indiana University Bloomington, Bloomington, IN, United States

**Keywords:** adult neuroplasticity, GABA receptors, glutamate receptors, developmental recapitulation, sensory deprivation

## Abstract

Although neural plasticity is now widely studied, there was a time when the idea of adult plasticity was antithetical to the mainstream. The essential stumbling block arose from the seminal experiments of Hubel and Wiesel who presented convincing evidence that there existed a critical period for plasticity during development after which the brain lost its ability to change in accordance to shifts in sensory input. Despite the zeitgeist that mature brain is relatively immutable to change, there were a number of examples of adult neural plasticity emerging in the scientific literature. Interestingly, some of the earliest of these studies involved visual plasticity in the adult cat. Even earlier, there were reports of what appeared to be functional reorganization in adult rat somatosensory thalamus after dorsal column lesions, a finding that was confirmed and extended with additional experimentation. To demonstrate that these findings reflected more than a response to central injury, and to gain greater control of the extent of the sensory loss, peripheral nerve injuries were used that eliminated ascending sensory information while leaving central pathways intact. Merzenich, Kaas, and colleagues used peripheral nerve transections to reveal unambiguous reorganization in primate somatosensory cortex. Moreover, these same researchers showed that this plasticity proceeded in no less than two stages, one immediate, and one more protracted. These findings were confirmed and extended to more expansive cortical deprivations, and further extended to the thalamus and brainstem. There then began a series of experiments to reveal the physiological, morphological and neurochemical mechanisms that permitted this plasticity. Ultimately, Mowery and colleagues conducted a series of experiments that carefully tracked the levels of expression of several subunits of glutamate (AMPA and NMDA) and GABA (GABAA and GABAB) receptor complexes in primate somatosensory cortex at several time points after peripheral nerve injury. These receptor subunit mapping experiments revealed that membrane expression levels came to reflect those seen in early phases of critical period development. This suggested that under conditions of prolonged sensory deprivation the adult cells were returning to critical period like plastic states, i.e., developmental recapitulation. Here we outline the heuristics that drive this phenomenon.

## Introduction

Although neural plasticity is now one of the most widely researched phenomena in the field, there was a time when the idea of adult plasticity was antithetical to the mainstream. The essential stumbling block was that the robust structural and functional effects of early disruptions of normal visual experience were not apparent in adult models. For example, Wiesel and Hubel (1963a) reported marked atrophy of cells in the deprived layers of the cat lateral geniculate nucleus (LGN) when monocular visual deprivation began early in life. Adult-onset monocular deprivation, on the other hand, had no effect on LGN cell size. Similarly, early visual deprivation resulted in a profound effect in striate cortex such that nearly all of the recorded cells responded only to inputs conveyed by the non-deprived eye but no such effect was found when the deprivation began in adulthood (Wiesel and Hubel, 1963b). Subsequently, Hubel and Wiesel (1970) extended these findings and identified “the period of susceptibility.” These studies provided convincing evidence that there existed a critical period for plasticity during visual system development after which the brain lost its ability to change in accordance to shifts in sensory input.

Despite the prevailing wisdom that mature brain is relatively immutable to change, there were a number of examples of adult neural plasticity emerging in the scientific literature. Interestingly, some of the earliest of these studies involved visual plasticity in the adult cat. A brief paper by Fiorentini and Maffei (1974) reported reduced binocularity in simple cells in adult cat visual cortex after the surgical immobilization of one eye, even with concurrent binocular deprivation (Maffei and Fiorentini, 1976). Other researchers (Brown and Salinger, 1979) reported the loss of X-cells in the layers of the adult cat LGN innervated by the immobilized eye following monocular paralysis, showing that adult neural plasticity could also be demonstrated in subcortical sites. A number of other examples of experience-dependent changes in adult visual system followed (e.g., Creutzfeldt and Heggelund, 1975; Hoffmann and Cynader, 1977; Salinger et al., 1977a,b, 1980a,b; Berlucchi et al., 1978a,b,c, 1979; Hoffmann and Holländer, 1978; Garraghty et al., 1982).

Even earlier, Wall and Egger (1971) reported functional reorganization in adult rat somatosensory thalamus after dorsal column lesions. Other experiments followed that showed plasticity in the dorsal spinal cord (e.g., Bausbaum and Wall, 1976; Wall, 1977), brainstem (e.g., Dostrovsky et al., 1976), and thalamus (e.g., Wall and Egger, 1971; Pollin and Albe-Fessard, 1979) after dorsal rhizotomies or dorsal column lesions. In these studies, cells in deafferented regions displayed abnormal receptive field properties that included responses to stimulation of intact peripheral pathways. Furthermore, it became apparent that this phenomenon was more than a transient response to deafferentation, as investigation of the temporal nature of these effects suggested that these changes could be both very acute (immediate) as well as chronic (e.g., Dostrovsky et al., 1976; Millar et al., 1976). Surprisingly (in retrospect), resistance to the possibility of adult neural plasticity remained strong.

## Plasticity in adult primate somatosensory cortex

**Figure 1:**
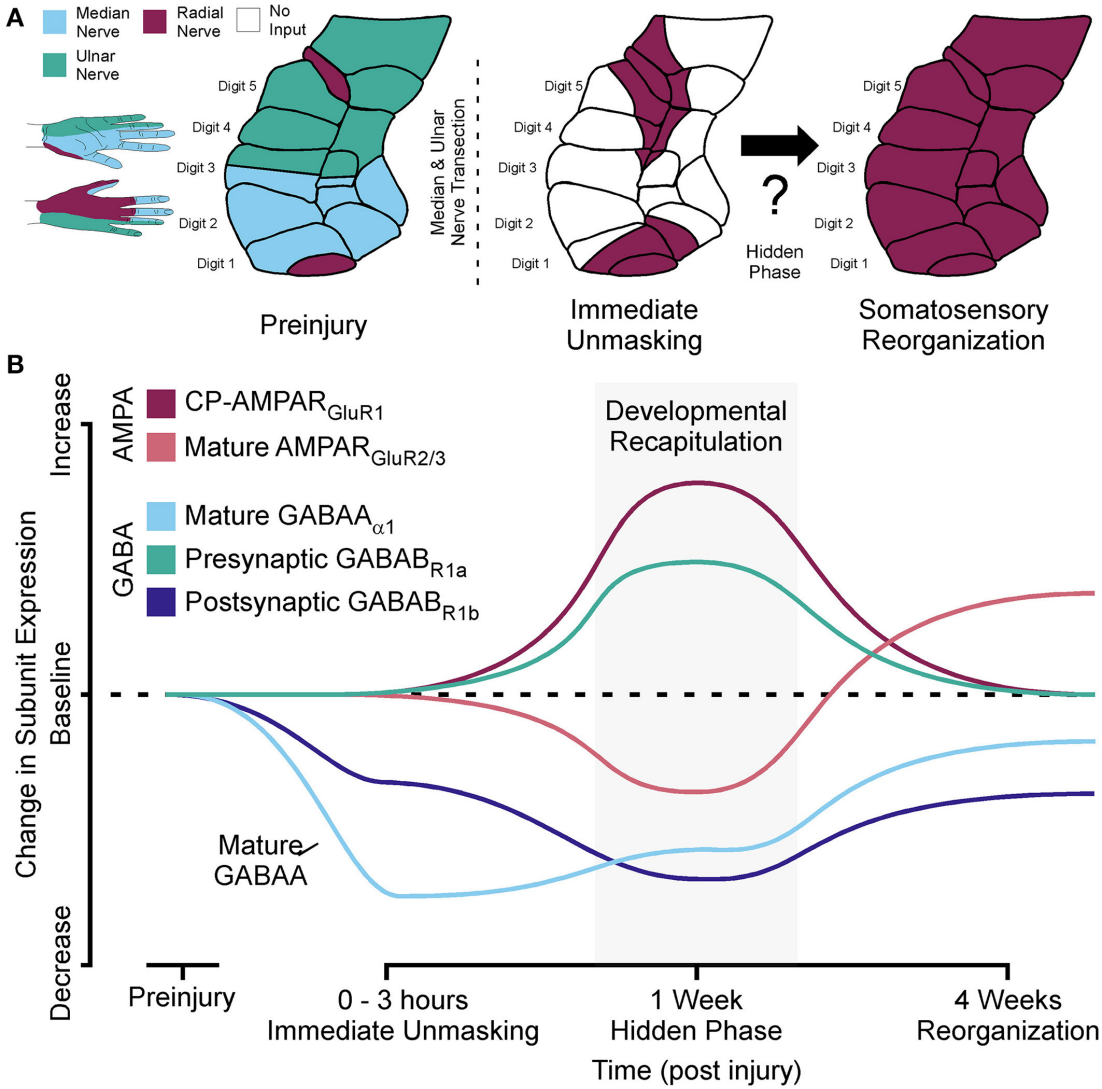
Developmental recapitulation: a hidden phase of somatosensory reorganization. **(A)** Left: Cartoon showing the innervation by the median, ulnar, and radial nerve inputs and corresponding receptive fields in area 3b cortex of the non-human primate hand region prior to injury. Right: Cartoon showing how the radial nerve inputs are immediately unmasked prior to complete reorganization of non-human primate area 3b hand representations after median and ulnar nerve transection. **(B)** Line diagram demonstrating how the shift in AMPA and GABA receptor subunit expressions reveal a previously hidden phase of adult somatosensory reorganization associated with the recapitulation of developmental receptor states.

In 1983, Merzenich et al. (1983a,b), reported on a series of seminal investigations that provided conclusive evidence that the topographic map of the body in adult primate somatosensory cortex could undergo substantial changes when parts of the map were deprived of their activating inputs *via* peripheral nerve transection. These experiments had two major advantages over the findings briefly discussed above. First, the transection of a peripheral nerve (the median nerve in these experiments) deprives a precise portion of the topographic map, eliminating any possible ambiguity as to the extent of the deafferentation. Second, these researchers used New World primates, such as the squirrel monkey *Saimiri Saimirinae* or owl monkey *Aotus Aotidae* as their subjects. These smaller primates, which descended from old world monkeys and apes about 40 million years ago, have brains that are relatively lissencephalic, and primary somatosensory cortex is exposed on the outer surface of the brain, rather than being buried in the central sulcus as it is in Old World primates and humans. Thus, the recording sites in the deprived portion of the topographic map could be unambiguously sited on photographs of the cortical surface as the primary somatosensory area (see Figure 1; Merzenich et al., 1983b). This latter fact made it possible to monitor the progression of the topographic reorganization over time after the nerve transection within individual subjects. These sequential mappings over time demonstrated that the reorganization proceeded in no less than two phases (see Churchill et al., 1998). Immediately following nerve transection, “new” inputs were recorded in restricted regions of the deprived patch of cortex. Over the following days to weeks, the second phase of reorganization proceeded, as the remaining areas of the deprived cortex became responsive to skin surfaces on the hand with intact innervation.

These ground-breaking discoveries engendered a number of new lines of research. Included among these were experiments that examined use-dependent alterations in cortical topography (e.g., Jenkins et al., 1990; Recanzone et al., 1992), in experiments that behaviorally controlled the tactile experience of the subjects. Allard et al. (1991) used digit syndactyly to show that when receptors adjacent digits were consistently coactivated because the digits were surgically fused, the normally discrete digit representation in primary somatosensory cortex became fused as well. Garraghty and Muja (1995) showed similar fusions in a monkey with a paralytic condition in one hand such that cortical neuronal receptive fields matched the aberrant pattern of skin surface coactivations that the paralysis produced. By labeling individual thalamocortical axonal arbors, the possible anatomical substrates supporting the plasticity were explored (e.g., Garraghty et al., 1989; Garraghty and Sur, 1990). These experiments showed that individual axonal arbors were larger than the grain of the topographic map, offering a means by which receptive fields could move across the cortex, as happens with nerve injury-induced reorganization. Potential neurochemical mechanisms were examined. Garraghty et al. (1991) used immunostaining for GABA to show reductions in the region of cortex that had undergone reorganization after nerve injury. Avendaño et al. (1995) showed that cholinergic mechanism were involved in the brain's response to sensory loss. Additional studies evaluated other patterns or extents of sensory loss (e.g., Wall et al., 1983; Merzenich et al., 1984; Garraghty and Kaas, 1991a; Garraghty et al., 1994). Observations of nerve injury-induced plasticity were also extended to subcortical levels (e.g., Garraghty and Kaas, 1991b; Faggin et al., 1997; Churchill et al., 2001).

The earlier work of Wall and colleagues (e.g., Merrill and Wall, 1972; Wall, 1977) characterized the immediate phase of plasticity as the “unmasking” of latent inputs. These were defined as peripheral nerve receptive fields that were normally suppressed by the dominant nerve inputs to these cortical areas (e.g., radial nerve receptive fields in median nerve cortical territory). When the dominant input was removed, these subordinate receptive fields were expressed or “unmasked.” Several lines of research subsequently offered confirmation for this idea. First, there were several reports of increases in the receptive field sizes of cortical neurons when inhibition within the cortex was blocked with bicuculline (e.g., Hicks and Dykes, 1983; Dykes et al., 1984; Alloway et al., 1989), indicating that “latent” inputs were available to cortical neurons. Second, using suprathreshold whole nerve stimulation, Schroeder et al. (1995) showed that latent inputs could be revealed in somatosensory cortex. In these experiments, latent radial, but not ulnar, nerve inputs were recorded in “median nerve cortex,” a finding that was consistent with the fact that the expansion of radial nerve-innervated skin surfaces accounted for most of the reorganization found in monkey cortex after median nerve transection (Merzenich et al., 1983a; Schroeder et al., 1997b; Myers et al., 2000). Finally, receptor autoradiographic (Wellman et al., 2002; Garraghty et al., 2006) and immunohistochemical experiments (Mowery et al., 2011) showed changes in GABA receptors that are consistent with a reduction in intracortical inhibition. Thus, the immediate topographic changes in the cortex after peripheral nerve injury appear to depend on the revelation of latent inputs that are normally under tonic inhibitory suppression.

The search for the mechanism(s) responsible for the protracted phase of reorganization was more challenging. At the simplest level, this stage of reorganization had to be due to either the sprouting of new connections, the strengthening of existing connections, or both. Anatomical studies examining the sizes of thalamocortical axonal arbors showed that the existing infrastructure was sufficient to permit the plasticity (Garraghty et al., 1989; Garraghty and Sur, 1990), suggesting that previously ineffective synapses were being strengthened. Motivated by the extensive literature involving glutamatergic NMDA receptor-dependent plasticity, experiments were conducted to investigate the possible contributions of these receptors to the topographic plasticity following peripheral nerve injury in adult monkeys. Not surprisingly, the immediate phase of reorganization proceeded whether NMDA receptors were blocked or not (Myers et al., 2000). The second stage of reorganization, on the other hand, was prevented if NMDA receptors were blocked (Garraghty and Muja, 1996). Thus, NMDA receptors were shown to be necessary for the “expression” of the second phase of cortical reorganization but not for its “maintenance.” Moreover, receptor autoradiography showed increases in AMPA glutamatergic receptors that correlated with the second stage of reorganization (Garraghty et al., 2006). Classic long-term potentiation (LTP) in the hippocampus had been shown previously to be NMDA receptor-dependent for its induction but not for its maintenance (e.g., Collingridge and Bliss, 1987). Furthermore, the maintenance of the LTP has been shown to involve the postsynaptic accumulation of AMPA receptors (e.g., Tocco et al., 1992; Maren et al., 1993; for a recent review, see Díaz-Alonzo and Nicoll, 2021). These obvious parallels between hippocampal LTP and nerve injury-induced topographic reorganization in primate somatosensory cortex have been previously addressed (Garraghty et al., 1998, 2006).

## Evidence for the recapitulation of developmental plasticity in adult somatosensory cortex after peripheral nerve injury

Despite their similarities, fundamental differences remained between hippocampal LTP and somatosensory plasticity in their routes of induction, longevity, and temporal progression. Most importantly was the transient nature of hippocampal LTP vs. the presumed permanence of the nerve injury-induced changes in the somatosensory cortex. These differences led to the consideration of other possibilities. Dykes and Lamour (1988) reported the intriguing finding that the majority of neurons in primary somatosensory cortex (in cats) had no receptive fields. That is, they could not be activated by peripheral stimulation. Subsequently, Warren and Dykes (1992) showed that a subset of these unresponsive neurons became responsive when glutamate was applied to the cortex iontophoretically, but nearly half of the recorded neurons remained unresponsive to peripheral stimulation. These findings raised the possibility that the large subset of neurons with no demonstrable peripheral receptive field became responsive during the second stage of reorganization in monkey cortex. Some support for this possibility was reported by Schroeder et al. (1997a) who showed that the blockade of GABA in the cortex (here, visual cortex) resulted in a marked increase in cortical excitability that could be reversed with the blockade of NMDA receptors. Intracortical measures of GABAA and GABAB receptors are found to be low as the second stage of reorganization proceeds (Garraghty et al., 2006). Moreover, this plasticity is prevented by NMDA receptor blockade (Garraghty and Muja, 1996). Thus, it seemed possible that increased excitability in the cortex mediated by NMDA receptors was a critical contributor in this plasticity.

When network activity drops drastically, as happens with a stroke, amputation, or nerve injury, synaptic excitatory and inhibitory receptor trafficking is dramatically altered in an experience dependent way (Arancibia-Cárcamo et al., 2009; Lussier et al., 2011). Under normal conditions, excitatory synapse maintenance is carried out through postsynaptic receptor trafficking of AMPA receptors containing largely Glur2/3 subunits (Tanaka et al., 2000). *In vitro*, when presynaptic glutamate release falls drastically (e.g., with tetrodotoxin application), cells increase excitability by trafficking calcium permeable forms of AMPA receptor (CP-AMPARs) to the synapse (Wierenga et al., 2005), CP-AMPARs are special types of receptors that gate calcium and drive NMDA-like processes that can induce LTP (e.g., Asrar et al., 2009). These GluR2 lacking calcium permeable AMPA receptors have been shown to play a major role in promoting circuit lability and metaplasticity (Clem and Huganir, 2010; Herry et al., 2010; Shepherd, 2012), and, thus, can enable potentiation at deprived synapses. This increase in lability occurs through the ability of the CP-AMPARs ability to gate calcium, thus giving them potentiating potential when NMDAR function is limited. This type of synaptic plasticity falls into the category of meta-plasticity, where neural activity can influence synaptic function at adjacent synapses over longer timelines. In fact, CP-AMPARs appear to play a significant role in activating silent synapses (Isaac et al., 1995; Liao et al., 1995). Silent synapses exist in developing systems prior to the onset of feed-forward activation in sensory systems. Here, primary inputs achieve dominance of the network while latent inputs remain muted. With adult-onset sensory deprivations, the latent inputs can be unmasked and the silent synapses activated through the CP-AMPARs.

As discussed above, an immediate unmasking of latent inputs occurs after a network wide reduction in dominant afferent drive with nerve injury (Merzenich et al., 1983b; Schroeder et al., 1997b; Myers et al., 2000). This unmasking is enabled by the removal of GABAA and GABAB receptors from synaptic sites of deprived networks (Wellman et al., 2002; Garraghty et al., 2006). In the weeks following this unmasking, these latent inputs come to reliably activate the deprived cortical region (Merzenich et al., 1983a,b; Garraghty and Kaas, 1991a; Schroeder et al., 1997a), and this process is NMDA receptor-dependent (Garraghty and Muja, 1996). NMDA receptor potentiation typically requires strong levels of feed forward activity to drive synaptic strengthening; however, activity levels are greatly diminished in a deprived network. This implied the existence of a previously hidden form of metaplasticity that could facilitate the onset of the NMDA dependent phase of sensory reorganization, which is active by 2 weeks post injury (see Cusick et al., 1990). Selective targeting of AMPA and GABA receptor subunits with immunocytochemical techniques at 1 week post injury in the deprived cortex showed receptor subunit configurations for AMPA (Mowery and Garraghty, 2009) and GABAA/GABAB receptors (Mowery et al., 2011) that were different from those associated with the immediate unmasking phase and the subsequent NMDA receptor-dependent phase of adult somatosensory plasticity. This pattern of receptor expression was more consistent with a recapitulation of “developmental” plasticity (Figure 1).

In developing networks, this pattern is associated with a reduced level of mature GluR2/3 subunit containing AMPAR in the active synapses that instead contain an elevated level of GluR1 subunits (Kumar et al., 2002; Eybalin et al., 2004; Ho et al., 2007; Whitney et al., 2008). In these immature networks, weak sensory afferent inputs (eyes, ears, and skin) can be potentiated through GluR1 containing AMPA receptor-mediated calcium gating that serves to un-silence the synapse and tag it for GluR2 containing AMPA receptor delivery and mature forms of NMDAR Hebbian strengthening. In the adult primate somatosensory cortex, similar changes to the expression of GluR1 and GluR2/3 subunits occurred shortly after peripheral nerve injury (Mowery and Garraghty, 2009) suggesting that GluR1 containing calcium permeable AMPARs might govern synaptic excitatory plasticity in cases where dominant excitatory inputs are severely reduced (injury) or lost (amputation). After sensory loss in the adult, a re-emergence of this mechanism could facilitate the synaptic strengthening of latent subordinate synaptic connections located in more distal regions of the dendritic trees of cortical neurons (see Churchill et al., 2004).

In an emerging sensory system, excitation and inhibition are skewed toward excitatory processes to allow the onset of peripheral input to engage synaptic strengthening mechanisms. In very immature neural networks, GABAergic synapses form first and are depolarizing until the chloride battery comes online (see Ben-Ari, 2002). The onset of glutamatergic feedforward activity begins the process. As the chloride transporter KCC2 matures, weak inhibitory hyperpolarization gradually emerges as the chloride reversal potential moves toward adult levels. This activity dependent step is vital for the progressive rebalancing of excitatory and inhibitory synapses toward their mature states (Cancedda et al., 2007). During development lowered inhibition serves an important purpose, as the lack of mature hyperpolarizing postsynaptic GABAA receptors increases the probability of postsynaptic depolarization and promotes CP-AMPA mediated potentiation. At the same time, the lack of functional postsynaptic GABAB receptors, which inhibit NMDA receptor activation, promotes NMDA induced strengthening of the synapses (see Otmakhova and Lisman, 2004). Presynaptic GABAB receptors; however, are functionally active during development. These autoreceptors regulate postsynaptic GABAergic signaling in the face of immature postsynaptic GABAergic synapses (McLean et al., 1996) that lack a functionally relevant population of GABAA receptors (Paysan et al., 1994). In network states where inhibitory tone has been reduced, presynaptic GABAB autoreceptors likely regulate GABAergic transmission.

**Figure 2:**
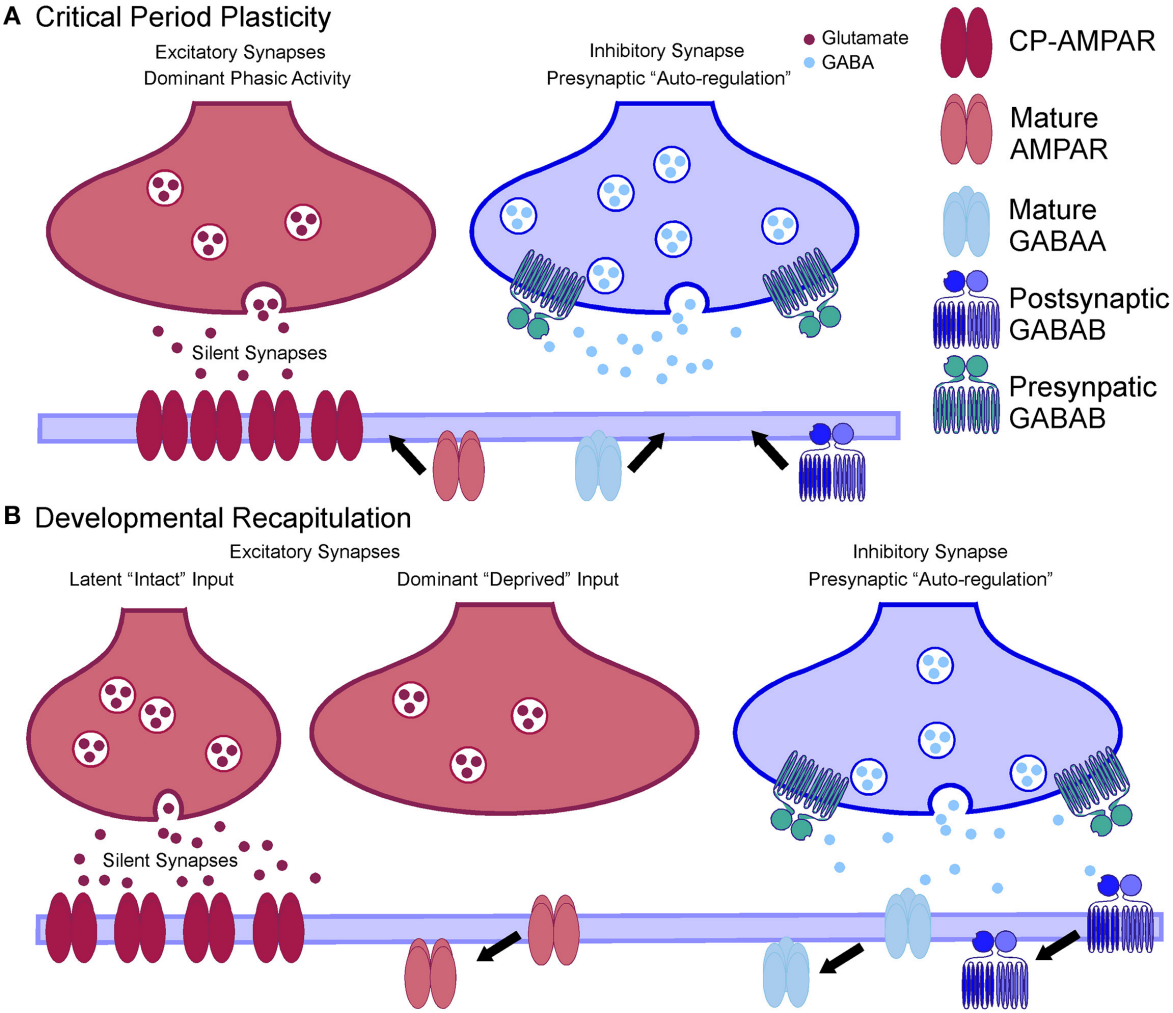
Parallels between critical period plasticity at developing synapses and developmental recapitulation at adult synapses. **(A)** Left: Cartoon showing calcium permeable AMPAR at silent excitatory synapses being activated and recruiting mature GluR2/3 containing AMPAR to the active dominant synapse. Right: Cartoon showing presynaptic GABAB autoregulation of inhibitory synapses prior to delivery of the mature postsynaptic GABAA and GABAB receptors, which are a hallmark of the closure of critical period plasticity. **(B)** Left: Cartoon showing the activation of calcium-permeable AMPAR at latent silent synapses after sensory deprivation of the normally dominant inputs. GluR2/3 containing AMPAR are removed from the deprived synapses until CP-AMPAR mediated processes can establish new “dominant” inputs. Right: Postsynaptic GABAB receptor autoregulation controls GABAergic inhibitory tone at synapses that have had the mature GABAA and GABAB receptors removed to promote activation of silent synapses.

In cases of sensory deprivation in the adult, a recapitulation of this postsynaptic inhibitory configuration as described above would again support the activation of silent latent synapses from the remaining intact peripheral nerves. The reduction in postsynaptic GluR2/3, GABAA, GABAB subunits, as well as the increase in GluR1 and presynaptic GABAB subunits found in adult primate somatosensory cortex 1 week after nerve injury (Mowery and Garraghty, 2009; Mowery et al., 2011) mirrors the conditions seen in developing networks (Figure 2). That is, the excitatory/inhibitory (E/I) tone is imbalanced toward excitation with low levels of active GABAA (Golshani et al., 1997; Paysan and Fritschy, 1998) and postsynaptic GABAB receptors (Fukuda et al., 1993; Fritschy et al., 1999) that are regulated by presynaptic GABAB receptors (McLean et al., 1996). The heightened excitatory state is only rebalanced to the mature E/I tone after active synapses are re-established by the still active latent inputs, which is a similar set of conditions these networks are exposed to when feedforward peripheral activity first emerges during development.

## Evidence for the recapitulation of developmental plasticity in other sensory and central systems after deprivation and injury

The onset of “adult-like” cortical inhibition is highly correlated with the closure of the critical period of plasticity in the visual cortex (e.g., Huang et al., 1999; Hensch, 2005) and auditory cortex (Mowery et al., 2015, 2019). After this period, both visual (Hubel and Wiesel, 1963; Berardi et al., 2000) and auditory systems become resistant to general changes in sensory input (Takesian et al., 2012; Mowery et al., 2016). However, drastic changes to sensory input comparable to somatosensory nerve injuries (e.g., retinal and cochlear denervation) induce retinotopic reorganization of the adult visual cortex (Kaas et al., 1990) and tonotopic reorganization in the adult auditory cortex (Schwaber et al., 1993; Eggermont, 2017). Furthermore, the reorganization phase occurs after an “unmasking” phase where latent intact inputs are immediately expressed in visual (e.g., Chino et al., 1992) and auditory cortex (e.g., Irvine and Rajan, 1997; Mossop et al., 2000). Thus, it seems plausible that the previously hidden phase of plasticity revealed in the somatosensory cortex exists for the visual and auditory systems as well.

A careful review of the literature in which data exist for intermediary time-points between unmasking and reorganization does provide some initial evidence that a brief window of developmental recapitulation opens. However, carefully designed research will be needed to confirm this (e.g., see Nahmani and Turrigiano, 2014 for review). A reduction of GABAergic inhibition is present at all three stages of reorganization so studies supporting this effect are not surprising. Thus, many studies have provided evidence of reduced GABAergic inhibition related to lowered expression or down-regulation of GABA subunits in the deprived ocular dominance column of the visual cortex (Hendry et al., 1994) or areas of the auditory neuraxis after cochlear ablation (e.g., inferior colliculus, Bledsoe Jr et al., 1995; Mossop et al., 2000) or denervation (e.g., auditory cortex, Balaram et al., 2019). To date, no studies have investigated the effect of adult onset visual or auditory sensory loss for either pre- or post-synaptic GABAB expression or function. On the other hand, a hallmark of the developmental plasticity is a reduction of the GluR2 containing AMPA receptors, which are replaced by calcium permeable homomeric GluR1 receptors. Both monocular deprivation and cochlear denervation lead to an acute reduction of GluR2 receptor in the deprived visual dominance column (Wong-Riley and Jacobs, 2002) and the inferior colliculus or auditory cortex (Balaram et al., 2019). Furthermore, an increase in phosphorylation of the GluR1 containing AMPAR (serine 845 site) accompanied the appearance of CP-AMPARs at synapses following visual deprivation (Goel et al., 2011). Direct studies of this phenomenon in the visual or auditory cortex have not been carried out as of yet, but there is evidence to support preliminary investigation. It is worth noting that similar evidence for the emergence of developmental plasticity has been reported after other forms of central nervous system injuries (Emery et al., 2003), such as ischemia (e.g., Gorter et al., 1997), spinal cord injury (e.g., Harel and Strittmatter, 2006), and epilepsy (e.g., Rivera et al., 2005). Together, these pieces of evidence from many brain regions provide the rationale to search for a universal neural mechanism governing this brief window of plasticity.

## The role of developmental recapitulation in the onset of maladaptive plasticity

Sensory deprivation during the critical period of development leads to persistent changes in sensory receptive fields (for review see Pedrosa et al., 2022). This can include massive reorganizations within a sensory modality or even across modalities such as when children are born deaf or blind (Sadato et al., 2002; Sathian, 2005). As we have outlined above, similar reorganizations happen in the adult networks when changes to dominant sensory inputs occur, but it is important to outline any possible differences between developmental plasticity in neonates and developmental recapitulation in adult neural networks. Topographic mapping in non-human primate neonates using microelectrode recordings (Krubitzer and Kaas, 1988) or fMRI (Arcaro et al., 2019) have shown that the cortical topographic map in infants are basically indistinguishable from those in older monkeys. Given this fact, it is perhaps not surprising that nerve transections performed on infant primates resulted in patterns of topographic reorganization very comparable to the map changes with adult-onset nerve injury (Wall et al., 1992a,b).

Unfortunately, no time course or acute mapping studies were carried out after the infant-onset nerve transections, so it cannot be known that the mechanisms involved in the map reorganizations from following early sensory loss are the same as those discussed above for adult-onset nerve transections. However, the comparability of the topographic maps in infant and adult primates (Krubitzer and Kaas, 1988; Arcaro et al., 2019) does suggest that similar neural mechanisms guide the neural response to deprivation and injury in neonates and adults. Therefore, the major difference between the two states of critical period plasticity and developmental recapitulation doesn't involve the plasticity mechanism, but the neural scaffolding that is available to harness this plasticity. In adults, nerve injuries are often accompanied by the emergence of chronic side effects that greatly lower quality of life. For example, after somatosensory injury, chronic pain and phantom sensations often emerge (Flor et al., 2006). In the auditory system, the onset of tinnitus (phantom auditory tones) accompanies recovery from auditory nerve/hair cell injury (Baguley, 2002). For the visual system, retinopathy can lead to reorganization that eventually causes visual field defects (Safran and Landis, 1999).

These reorganizations are thought to be the consequence of maladaptive plasticity, and the etiological culprit could be related to the re-emergence of critical period-like states that allow aberrant functional connections to form between synapses deprived of their dominant inputs and adjacent intact functional synapses. This could offer an important clue toward the development of classes of drugs targeting the calcium permeable AMPARs or GABARs at these sensitive points to prevent this maladaptive plasticity from taking hold. Being able to evoke developmental recapitulation in the adult nervous system outside of reorganizing injuries would also be an interesting line of research toward the development of effective interventions for chronic nerve injuries that are largely untreatable. In the auditory system, exposure to auditory noise, has been suggested to “re-open” the auditory critical period (Zhou et al., 2011). Bavelier et al. (2010) used a pharmacological approach to re-induce the critical-period and treat amblyopia. Perhaps a similar approach using tactile stimulation or neuromodulators could be explored toward the treatment of nerve injury induced somatosensory disorders.

## Conclusion

Our first foray into the issue of adult somatosensory plasticity examined the sizes of thalamocortical axonal arbors, as this was an essential piece of information needed to guide subsequent experiments. If thalamocortical axonal arbors precisely terminated in topographically appropriate patches of cortex, the sprouting of new connections would seemingly be required to move receptive fields across the cortex. As it turned out, we found that the axonal arbors were larger than the zones of cortex where their receptive fields were manifested. This “degenerate” anatomy (Edelman, 1987) clearly suggested that subthreshold inputs existing in the cortex gained strength during the reorganizational process. Thus, research in the field came to center on the mechanism(s) by which this strengthening occurred. Experiments targeting GABAergic mechanisms revealed the contribution of this neurochemical system to the immediate unmasking that followed the sensory loss. The relaxation of feedforward inhibition also permitted glutamatergic mechanisms to contribute to the latter phases of reorganization. With the finding that glutamatergic NMDA receptors are necessary for the latter stages of reorganization, we began view the peripheral nerve transection paradigm as a platform for studying adult neural plasticity *per se*, and not merely a feature of the somatosensory system. Ultimately, in our view, this nerve injury model in adult primates has revealed mechanisms of neural change that apply broadly across the brain, and the recapitulation of developmental plasticity is an important feature of experience-dependent adult plasticity.

## Data availability statement

The original contributions presented in the study are included in the article/supplementary material, further inquiries can be directed to the corresponding author.

## Author contributions

Both authors listed have made a substantial, direct, and intellectual contribution to the work and approved it for publication.

## References

[B1] Allard T. Clark S. A. Jenkins W. M. Merzenich M. M. (1991). Reorganization of somatosensory area 3b representations in adult owl monkeys after digit syndactyly. J. Neurophysiol. 66, 1048–1058. 10.1152/jn.1991.66.3.1048 1753275

[B2] Alloway K. D. Rosenthal P. Burton H. (1989). Quantitative measurements of receptive field changes during antagonism of GABAergic transmission in primary somatosensory cortex. Exp. Brain Res.78, 514–532. 10.1007/BF00230239 2612595

[B3] Arancibia-Cárcamo I. L. Yuen E. Y Muir J. Lumb M. J. Michels G. Saliba R. S. Smart T. G. . (2009). Ubiquitin-dependent lysosomal targeting of GABA(A) receptors regulates neuronal inhibition. Proc. Natl. Acad. Sci. USA 106, 17552–17557. 10.1073/pnas.0905502106 19815531 PMC2762659

[B4] Arcaro M. J. Schade P. F. Livingstone M.S. (2019). Body map proto-organization in newborn macaques. Proc. Natl. Acad. Sci. U S A. 116, 24861–24871. 10.1073/pnas.1912636116 31732670 PMC6900594

[B5] Asrar S. Zhou Z. Ren W. Jia Z. (2009). Ca(2+) permeable AMPA receptor induced long-term potentiation requires PI3/MAP kinases but not Ca/CaM-dependent kinase II. PLoS ONE 4, e4339. 10.1371/journal.pone.0004339 19190753 PMC2629531

[B6] Avendaño C. Umbriaco D. Dykes R. W. Descarries L. (1995). Decrease and long-term recovery of choline acetyltransferase immunoreactivity in adult cat somatosensory cortex after peripheral nerve transections. J. Comp. Neurol. 354, 321–332. 10.1002/cne.903540302 7541804

[B7] Baguley D. M. (2002). Mechanisms of tinnitus. Br. Med. Bull. 63, 195–212. 10.1093/bmb/63.1.195 12324394

[B8] Balaram P. Hackett T. A. Polley D. B. (2019). Synergistic transcriptional changes in AMPA and GABAA receptor genes support compensatory plasticity following unilateral hearing loss. Neuroscience 407, 108–119. 10.1016/j.neuroscience.2018.08.023 30176318 PMC6395571

[B9] Bausbaum A. I. Wall P. D. (1976). Chronic changes in the response of cells in adult cat dorsal horn following partial deafferentation. The appearance of responding cells in a previously non-responsive region. Brain Res. 116, 181–204. 10.1016/0006-8993(76)90899-4 974771

[B10] Bavelier D. Levi D. M. Li R. W. Dan Y. Hensch T. K. (2010). Removing brakes on adult brain plasticity: from molecular to behavioral interventions. J. Neurosci. 30, 14964–14971. 10.1523/JNEUROSCI.4812-10.2010 21068299 PMC2992973

[B11] Ben-Ari Y. (2002). Excitatory actions of gaba during development: the nature of the nurture. Nat. Rev. Neurosci. 3, 728–739. 10.1038/nrn920 12209121

[B12] Berardi N. Pizzorusso T. MaVei L. (2000). Critical periods during sensory development. Curr. Opin. Neurobiol. 10, 138–145. 10.1016/S0959-4388(99)00047-1 10679428

[B13] Berlucchi G. Buchtel E. Marzi C. A. Mascetti G. G. Simoni A. (1978a). Effects of experience on interocular transfer of pattern discriminations in split-chiasm and split-brain cats. J. Comp. Physiol. Psych. 92, 532–543. 10.1037/h0077482 681568

[B14] Berlucchi G. Buchtel H. A. Lepore F. (1978b). Successful interocular transfer of visual pattern discriminations in split-chiasm cats with section of the intertectal and posterior commissures. Physiol. Behav. 20, 331–338. 10.1016/0031-9384(78)90228-7 748942

[B15] Berlucchi G. Sprague J. M. Antonini A. Simoni A. (1979). Learning and interhemispheric transfer of visual pattern discriminations following unilateral suprasylvian lesions in split-chiasm cats. Exp. Brain Res. 34, 551–574. 10.1007/BF00239149 421761

[B16] Berlucchi G. Sprague J. M. Lepore F. Mascetti G. G. (1978c). Effects of lesions of areas 17, 18 and 19 on interocular transfer of pattern discriminations in split-chiasm cats. Exp. Brain Res. 31, 275–297. 10.1007/BF00237604 631243

[B17] Bledsoe S. C. Jr Nagase S. Miller J. M. Altschuler R. A. (1995). Deafness-induced plasticity in the mature central auditory system. Neuroreport 7, 225–229. 10.1097/00001756-199512000-00054 8742457

[B18] Brown D. L. Salinger W. L. (1979). Loss of X cells in the lateral geniculate nucleus with monocular paralysis: neural plasticity in the adult cat. Science 189, 1011–1012. 10.1126/science.1220007 1220007

[B19] Cancedda L. Fiumelli H. Chen K. Poo M. (2007). Excitatory GABA action is essential for morphological maturation of cortical neurons in vivo. J. Neurosci. 27, 5224–5235. 10.1523/JNEUROSCI.5169-06.2007 17494709 PMC6672363

[B20] Chino Y. M. Kaas J. H. Smith E. L. 3rd Langston A. L. Cheng H. (1992). Rapid reorganization of cortical maps in adult cats following restricted deafferentation in retina. Vision Res. 32, 789–796. 10.1016/0042-6989(92)90021-A 1604848

[B21] Churchill J. D. Arnold L. L. Garraghty P. E. (2001). Somatotopic reorganization in the brainstem and thalamus following peripheral nerve injury in adult primates. Brain Res. 910, 142–152. 10.1016/S0006-8993(01)02703-2 11489264

[B22] Churchill J. D. Muja N. Myers W. A. Besheer J. Garraghty P. E. (1998). Somatotopic consolidation: a third phase of reorganization after peripheral nerve injury in adult squirrel monkeys. Exp. Brain Res. Trends Neurosci. 118, 189–196. 10.1007/s002210050271 9547087

[B23] Churchill J. D. Tharp J. A. Wellman C. L. Sengelaub D. R. Garraghty P. E. (2004). Morphological correlates of injury-induced reorganization in primate somatosensory cortex. BMC Neurosci. 5, 43. 10.1186/1471-2202-5-43 15533258 PMC529444

[B24] Clem R. L. Huganir R. L. (2010). Calcium-permeable AMPA receptor dynamics mediate fear memory erasure. Science. 330, 1108–1112. 10.1126/science.1195298 21030604 PMC3001394

[B25] Collingridge G. L. Bliss T. V. P. (1987). NMDA receptors—their role in long-term potentiation. Trends Neurosci.10, 288–293. 10.1016/0166-2236(87)90175-5

[B26] Creutzfeldt O. D. Heggelund P. (1975). Neural plasticity in visual cortex of adult cats after exposure to visual patterns. Science 188, 1025–1027. 10.1126/science.1145187 1145187

[B27] Cusick C. G. Wall J. T. Whiting J. H. Jr Wiley R. G. (1990). Temporal progression of cortical reorganization following nerve injury. Brain Res. 537, 355–358. 10.1016/0006-8993(90)90385-O 2085786

[B28] Díaz-Alonzo J. Nicoll R. A. (2021). AMPA receptor trafficking and LTP: carboxy-termini, amino-termini and TARPS. Neuropharmcology 197, 108710. 10.1016/j.neuropharm.2021.108710 34271016 PMC9122021

[B29] Dostrovsky J. O. Millar J. Wall P. D. (1976). The immediate shift of afferent drive of dorsal column nucleus cells following deafferentation: a comparison of acute and chronic deafferentation in gracile nucleus and spinal cord. Exp. Neurol. 52, 480–495. 10.1016/0014-4886(76)90219-3 954919

[B30] Dykes R. Lamour Y. (1988). Neurons without demonstrable receptive fields outnumber neurons having receptive fields in samples from the somatosensory cortex of anesthetized or paralyzed cats and rats. Brain Res.440, 133–143. 10.1016/0006-8993(88)91165-1 3359202

[B31] Dykes R. W. Landry P. Metherate R. Hicks T. P. (1984). Functional role of GABA in cat primary somatosensory cortex: shaping receptive fields of cortical neurons. J. Neurophysiol. 52, 1066–1093. 10.1152/jn.1984.52.6.1066 6151590

[B32] Edelman G. M. (1987). Neural Darwinism: The Theory of Neuronal Group Selection. New York, NY: Basic Books Inc. 10.1126/science.240.4860.1802 17842436

[B33] Eggermont J. J. (2017). Acquired hearing loss and brain plasticity. Hear. Res. 343, 176–190. 10.1016/j.heares.2016.05.008 27233916

[B34] Emery D. L. Royo N. C. Fischer I. Saatman K. E. McIntosh T. K. (2003). Plasticity following injury to the adult central nervous system: is recapitulation of a developmental state worth promoting? J. Neurotrauma. 20, 1271–1292. 10.1089/089771503322686085 14748977

[B35] Eybalin M. Caicedo A. Renard N. Ruel J. Puel J. L. (2004). Transient Ca2+-permeable AMPA receptors in postnatal rat primary auditory neurons. Eur. J. Neurosci. 20, 2981–2989. 15579152 10.1111/j.1460-9568.2004.03772.x

[B36] Faggin B. M. Nguyen K. T. Nicolelis M. A. L. (1997). Immediate and simultaneous sensory reorganization at cortical and subcortical levels of the somatosensory system. Proc. Natl. Acad. Sci. USA 94, 9428–9433. 10.1073/pnas.94.17.9428 9256499 PMC23207

[B37] Fiorentini A. Maffei L. (1974). Change of binocular properties of the simple cells of the cortex in adult cats following immobilization of one eye. Vision Res. 14, 217–218. 10.1016/0042-6989(74)90104-7 4818692

[B38] Flor H. Nikolajsen L. Staehelin Jensen T. (2006). Phantom limb pain: a case of maladaptive CNS plasticity? Nat. Rev. Neurosci. 7, 873–881. 10.1038/nrn1991 17053811

[B39] Fritschy J. M. Meskenaite V. Weinmann O. Honer M. Benke D. Mohler H. (1999). GABAB-receptor splice variants GB1a and GB1b in rat brain: developmental regulation, cellular distribution and extrasynaptic localization. Eur. J. Neurosci. 11, 761–768. 10.1046/j.1460-9568.1999.00481.x 10103070

[B40] Fukuda A. Mody I. Prince D. A. (1993). Differential ontogenesis of presynaptic and postsynaptic GABAB inhibition in rat somatosensory cortex. J. Neurophysiol. 70, 448–452. 10.1152/jn.1993.70.1.448 8395587

[B41] Garraghty P. E. Arnold L. L. Wellman C. L. Mowery T. M. (2006). Receptor autoradiographic correlates of deafferentation-induced reorganization in adult primate somatosensory cortex. J. Comp. Neurol. 497, 636–645. 10.1002/cne.21018 16739196 PMC4139035

[B42] Garraghty P. E. Churchill J. D. Banks M. K. (1998). Adult neural plasticity: similarities between two paradigms. Curr. Dir. Psych. Sci. 7, 87–91. 10.1111/1467-8721.ep107746616348606

[B43] Garraghty P. E. Hanes D. P. Florence S. L. Kaas J. H. (1994). Pattern of peripheral deafferentation predicts reorganizational limits in adult primate somatosensory cortex. Somatosens. Motor Res. 11, 109–117. 10.3109/08990229409028864 7976005

[B44] Garraghty P. E. Kaas J. H. (1991a). Largescale functional reorganization in adult monkey cortex after peripheral nerve injury. Proc. Natl. Acad. Sci. USA 88, 69766980. 10.1073/pnas.88.16.6976 1871112 PMC52216

[B45] Garraghty P. E. Kaas J. H. (1991b). Functional reorganization in adult monkey thalamus after peripheral nerve injury. NeuroReports 2, 747–750. 10.1097/00001756-199112000-00004 1793816

[B46] Garraghty P. E. LaChica E. A. Kaas J. H. (1991). Injuryinduced reorganization of somatosensory cortex is accompanied by reductions in GABA staining. Somatosens. Motor Res. 8, 347–354. 10.3109/08990229109144757 1667058

[B47] Garraghty P. E. Muja N. (1995). Possible usedependent changes in adult primate somatosensory cortex. Brain Res.686, 119–121. 10.1016/0006-8993(95)00506-L 7583265

[B48] Garraghty P. E. Muja N. (1996). NMDA receptors and plasticity in adult primate somatosensory cortex. J. Comp. Neurol. 367, 319–326. 10.1002/(SICI)1096-9861(19960401)367:2<319::AID-CNE12>3.0.CO;2-L 8708013

[B49] Garraghty P. E. Pons T. P. Sur M. Kaas J. H. (1989). The arbors of axons terminating in middle cortical layers of somatosensory area 3b in owl monkeys. Somatosens. Motor Res. 6, 401411. 10.3109/08990228909144683 2756803

[B50] Garraghty P. E. Salinger W. L. MacAvoy M. G. Schroeder C. E. Guido W. (1982). The shift in X/Y ratio after chronic monocular paralysis: a binocularly mediated, barbituratesensitive effect in the adult lateral geniculate nucleus. Exp. Brain Res. 47, 301308. 10.1007/BF00239390 7117455

[B51] Garraghty P. E. Sur M. (1990). The morphology of single intracellularly stained axons terminating in area 3b of macaque monkeys. J. Comp. Neurol. 294, 583593. 10.1002/cne.902940406 2341626

[B52] Goel A. Xu L. Snyder K. P. Song L. Goenaga-Vazquez Y. Megill A. . (2011). Phosphorylation of AMPA receptors is required for sensory deprivation-induced homeostatic synaptic plasticity. PLoS ONE. 6, e18264. 10.1371/journal.pone.0018264 21483826 PMC3069067

[B53] Golshani P. Truong H. Jones E. G. (1997). Developmental expression of GABA(A) receptor subunit and GAD genes in mouse somatosensory barrel cortex. J. Comp. Neurol. 383, 199–219. 10.1002/(SICI)1096-9861(19970630)383:2<199::AID-CNE7>3.0.CO;2-W 9182849

[B54] Gorter J. A. Petrozzino J. J. Aronica E. M. Rosenbaum D. M. Opitz T. Bennett M. V. . (1997). Global ischemia induces downregulation of Glur2 mRNA and increases AMPA receptor-mediated Ca^2+^ influx in hippocampal CA1 neurons of gerbil. J. Neurosci. 17, 6179–6188. 10.1523/JNEUROSCI.17-16-06179.1997 9236229 PMC6568367

[B55] Harel N. Y. Strittmatter S. M. (2006). Can regenerating axons recapitulate developmental guidance during recovery from spinal cord injury? Nat. Rev. Neurosci. 7, 603–616. 10.1038/nrn1957 16858389 PMC2288666

[B56] Hendry S. H Huntsman M. M. Viñuela A. Möhler H. de Blas A. L. Jones E. G. (1994). GABAA receptor subunit immunoreactivity in primate visual cortex: distribution in macaques and humans and regulation by visual input in adulthood. J. Neurosci. 14, 2383–2401. 10.1523/JNEUROSCI.14-04-02383.1994 8158275 PMC6577113

[B57] Hensch T. K. (2005). Critical period plasticity in local cortical circuits. Nat. Rev. Neurosci. 6, 877–888. 10.1038/nrn1787 16261181

[B58] Herry C. Ferraguti F. Singewald N. Letzkus J. J. Ehrlich I. Lüthi A. (2010). Neuronal circuits of fear extinction. Eur. J. Neurosci. 31, 599–612. 10.1111/j.1460-9568.2010.07101.x 20384807

[B59] Hicks T. P. Dykes R. W. (1983). Receptive field size for certain neurons in primary somatosensory cortex is determined by GABA-mediated intracortical inhibition. Brain Res. 274, 160–164. 10.1016/0006-8993(83)90533-4 6137268

[B60] Ho M. T. Pelkey K. A. Topolnik L. Petralia R. S. Takamiya K. Xia J. . (2007). Developmental expression of Ca2+-permeable AMPA receptors underlies depolarizationinduced long-term depression at mossy fiber CA3 pyramid synapses. J. Neurosci. 27, 11651–11662. 10.1523/JNEUROSCI.2671-07.2007 17959808 PMC6673239

[B61] Hoffmann K.-P. Holländer H. (1978). Physiological and morphological changes in cells of the lateral geniculate nucleus in monocularly-deprived and reverse-sutured cats. J. Comp. Neurol. 177, 145–158. 10.1002/cne.901770110 618437

[B62] Hoffmann K. P. Cynader M. (1977). Functional aspects of plasticity in the visual system of adult cats after early monocular deprivation. Philos. Trans. B 278, 411–424. 10.1098/rstb.1977.0051 19792

[B63] Huang Z. J. Kirkwood A. Pizzorusso T. Porciatti V. Morales B. Bear M. F. . (1999). BDNF regulates the maturation of inhibition and the critical period of plasticity in mouse visual cortex. Cell 98, 739–755. 10.1016/S0092-8674(00)81509-3 10499792

[B64] Hubel D. H. Wiesel T. N. (1963). Receptive fields of cells in striate cortex of very young, visually inexperienced kittens. J. Neurophysiol. 26, 994–1002. 10.1152/jn.1963.26.6.994 14084171

[B65] Hubel D. H. Wiesel T. N. (1970). The period of susceptibility to the physiological effects of unilateral eye closure in kittens. J. Physiol. 206, 419–436. 10.1113/jphysiol.1970.sp009022 5498493 PMC1348655

[B66] Irvine D. R. Rajan R. (1997). Injury-induced reorganization of frequency maps in adult auditory cortex: the role of unmasking of normally-inhibited inputs. Acta Otolaryngol. Suppl. 532, 39–45. 10.3109/00016489709126143 9442843

[B67] Isaac J. T. Nicoll R. A. Malenka R. C. (1995). Evidence for silent synapses: implications for the expression of LTP. Neuron. 15, 427–434. 10.1016/0896-6273(95)90046-2 7646894

[B68] Jenkins W. M. Merzenich M. M. Ochs M. T. Allard T. Guic-Robles E. (1990). Functional reorganization of primary somatosensory cortex in adult owl monkeys after behaviorally controlled tactile stimulation. J. Neurophysiol. 63, 82–104. 10.1152/jn.1990.63.1.82 2299388

[B69] Kaas J. H. Krubitzer L. A. Chino Y. M. Langston A. L. Polley E. H. Blair N. (1990). Reorganization of retinotopic cortical maps in adult mammals after lesions of the retina. Science 248, 229–231. 10.1126/science.2326637 2326637

[B70] Krubitzer L. A. Kaas J. H. (1988). Responsiveness and somatotopic organization of anterior parietal field 3b and adjoining cortex in newborn and infant monkeys. Somatosens Mot. Res. 6, 179–205. 10.3109/08990228809144673 3242345

[B71] Kumar S. S. Bacci A. Kharazia V. Huguenard J. R. (2002). A developmental switch of AMPA receptor subunits in neocortical pyramidal neurons. J. Neurosci. 22, 3005–3015. 10.1523/JNEUROSCI.22-08-03005.2002 11943803 PMC6757523

[B72] Liao D. Hessler N. A. Malinow R. (1995). Activation of postsynaptically silent synapses during pairing-induced LTP in CA1 region of hippocampal slice Nature 375, 400–404. 10.1038/375400a0 7760933

[B73] Lussier M. P. Nasu-Nishimura Y. Roche K. W. (2011). Activity-dependent ubiquitination of the AMPA receptor subunit GluA2. J. Neurosci. 31, 3077–3081. 10.1523/JNEUROSCI.5944-10.2011 21414928 PMC3081723

[B74] Maffei L. Fiorentini A. (1976). Asymmetry of motility of the eyes and changes in binocular properties of cortical cells in adult cats. Brain Res. 105, 73–78. 10.1016/0006-8993(76)90923-9 1252959

[B75] Maren S. Tocco G. Baudry M. Thompson R. F. (1993). Postsynaptic factors in the expression pf long-term potentiation (LTP): increased glutamate receptor binding following LTP induction in vivo. Proc. Natl. Acad. Sci. USA 90, 9654–9658. 10.1073/pnas.90.20.9654 8415757 PMC47628

[B76] McLean H. A. Caillard O. Khazipov R. Ben-Ari Y. Gaiarsa J. L. (1996). Spontaneous release of GABA activates GABAB receptors and controls network activity in the neonatal rat hippocampus. J. Neurophysiol. 76, 1036–1046. 10.1152/jn.1996.76.2.1036 8871218

[B77] Merrill E. G. Wall P. D. (1972). Factors forming the edge of a receptive field. The presence of relatively ineffective afferents. J. Physiol. 226, 825–846. 10.1113/jphysiol.1972.sp010012 4637631 PMC1331179

[B78] Merzenich M. M. Kaas J. H. Nelson R. J. Sur M. Felleman D. (1983a). Topographic reorganization of somatosensory cortical areas 3b and 1 in adult monkeys following restricted deafferentation. Neuroscience 8, 33–55. 10.1016/0306-4522(83)90024-6 6835522

[B79] Merzenich M. M. Kaas J. H. Wall J. T. Sur M. Nelson R. J. Felleman D. J. (1983b). Progression of change following median nerve section in the cortical representation of the hand in areas 3b and 1 in adult owl and squirrel monkeys. Neuroscience 10, 639–665. 10.1016/0306-4522(83)90208-7 6646426

[B80] Merzenich M. M. Nelson R. J. Stryker M. P. Cynader M. S. Schoppmann A. Zook J. M. (1984). Somatosensory cortical map changes following digit amputation in adult monkeys. J. Comp. Neurol. 224, 591–605. 10.1002/cne.902240408 6725633

[B81] Millar J. Bausbaum A. I. Wall P. D. (1976). Restructuring of the somatotopic map and appearance of abnormal neuronal activity in the gracile nucleus after partial deafferentation. Exp. Neurol. 50, 658–672. 10.1016/0014-4886(76)90035-2 1253869

[B82] Mossop J. E. Wilson M. J. Caspary D. M. Moore D. R. (2000). Down-regulation of inhibition following unilateral deafening. Hear. Res. 147, 183–187. 10.1016/S0378-5955(00)00054-X 10962184

[B83] Mowery T. M. Caras M. L. Hassan S. I. Wang D. J. Dimidschstein J. Fishell G. . (2019). Preserving inhibition during developmental hearing loss rescues auditory learning and perception. J. Neurosci. 39, 8347–8361. 10.1523/JNEUROSCI.0749-19.2019 31451577 PMC6794918

[B84] Mowery T. M. Garraghty P. E. (2009). Nerve-injury induced changes to GluR1 and GluR2/3 sub-unit expression in the area 3b of adult squirrel monkeys: developmental recapitulation. Front. Syst. Neurosci. 3, 1–7. 10.3389/neuro.06.001.2009 19212458 PMC2638550

[B85] Mowery T. M. Kotak V. C. Sanes D. H. (2016). The onset of visual experience gates auditory cortex critical periods. Nat. Commun. 7, 10416. 10.1038/ncomms10416 26786281 PMC4736048

[B86] Mowery T. M. Sarin R. M. Elliott K. S. Garraghty P. E. (2011). Nerve injury-induced changes in GABAA and GABAB sub-unit expression in area 3b and cuneate nucleus of adult squirrel monkeys: further evidence of developmental recapitulation. Brain Res. 1415, 63–75. 10.1016/j.brainres.2011.07.066 21880301

[B87] Mowery T. M. Sarin R. M. Kostylev P. V. Garraghty P. E. (2015). Differences in AMPA and GABAA/B receptor subunit expression between the chronically reorganized cortex and brainstem of adult squirrel monkeys. Brain Res. 1611, 44–55. 10.1016/j.brainres.2015.03.010 25791620 PMC4441862

[B88] Myers W. A. Churchill J. D. Muja N. Garraghty P. E. (2000). The role of NMDA receptors in adult primate cortical somatosensory plasticity. J. Comp. Neurol. 418, 373–382. 10.1002/(SICI)1096-9861(20000320)418:4<373::AID-CNE1>3.0.CO;2-F 10713567

[B89] Nahmani M. Turrigiano G. G. (2014). Adult cortical plasticity following injury: Recapitulation of critical period mechanisms? Neuroscience. 283, 4–16. 10.1016/j.neuroscience.2014.04.029 24791715 PMC4216647

[B90] Otmakhova N. A. Lisman J. E. (2004). Contribution of Ih and GABAB to synaptically induced afterhyperpolarizations in CA1: a brake on the NMDA response. J. Neurophysiol. 92, 2027–2039. 10.1152/jn.00427.2004 15163674

[B91] Paysan J. Bolz J. Mohler H. Fritschy J. M. (1994). GABAA receptor alpha 1 subunit, an early marker for area specification in developing rat cerebral cortex. J. Comp. Neurol. 350, 133–149. 10.1002/cne.903500110 7860797

[B92] Paysan J. Fritschy J. M. (1998). GABAA-receptor subtypes in developing brain. Actors or spectators? Perspect. Dev. Neurobiol. 5, 179–192. 9777635

[B93] Pedrosa L. R. Coimbra G. S. Corrêa M. G. Dias I. A. Bahia C. P. (2022). Time window of the critical period for neuroplasticity in S1, V1, and A1 sensory areas of small rodents: a systematic review. Front. Neuroanat. 16, 763245. 10.3389/fnana.2022.763245 35370567 PMC8970055

[B94] Pollin B. Albe-Fessard D. (1979). Organization of somatic thalamus in monkeys with and without section of dorsal spinal tracts. Brain Res.173, 431–449. 10.1016/0006-8993(79)90240-3 114272

[B95] Recanzone G. H. Merzenich M. M. Jenkins W. M. (1992). Frequency discrimination training engaging a restricted skin surface results in an emergence of a cutaneous response zone in cortical area 3a. J. Neurophysiol. 67, 1057–1070. 10.1152/jn.1992.67.5.1057 1597697

[B96] Rivera C. Voipio J. Kaila K. (2005). Two developmental switches in GABAergic signalling: the K+-Cl- cotransporter KCC2 and carbonic anhydrase CAVII. J. Physiol. 562, 27–36. 10.1113/jphysiol.2004.077495 15528236 PMC1665491

[B97] Sadato N. Okada T. Honda M. Yonekura Y. (2002). Critical period for cross-modal plasticity in blind humans: a functional MRI study. Neuroimage 16, 389–400. 10.1006/nimg.2002.1111 12030824

[B98] Safran A. B. Landis T. (1999). From cortical plasticity to unawareness of visual field defects. J. Neuroophthalmol. 19, 84–88. 10.1097/00041327-199906000-00002 10380128

[B99] Salinger W. L. Garraghty P. E. MacAvoy M. G. Hooker L. F. (1980b). Sensitivity of the mature lateral geniculate nucleus to components of monocular paralysis. Brain Res. 187, 307320. 10.1016/0006-8993(80)90205-X 7370732

[B100] Salinger W. L. Garraghty P. E. Schwartz M. A. (1980a). Response of the mature lateral geniculate nucleus to monocular paralysis: contributions of nonretinal and retinal components. Brain Res. 192, 255260. 10.1016/0006-8993(80)91025-2 7378783

[B101] Salinger W. L. Schwartz M. A. Wilkerson P. R. (1977a). Selective cell loss in the lateral geniculate nucleus of adult cats following binocular lid suture. Brain Res. 130, 81–88. 10.1016/0006-8993(77)90843-5 884521

[B102] Salinger W. L. Schwartz M. A. Wilkerson P. R. (1977b). Selective loss of lateral geniculate cells in the adult cat after chronic monocular paralysis. Brain Res. 125, 257–263. 10.1016/0006-8993(77)90619-9 851878

[B103] Sathian K. (2005). Visual cortical activity during tactile perception in the sighted and the visually deprived. Dev. Psychobiol. 46, 279–286. 10.1002/dev.20056 15772968

[B104] Schroeder C. E. Javitt D. C. Steinschneider M. Mehta A. D. Givre S. J. Vaughan H. G. Jr . (1997a). N-methyl-D-aspartate enhancement of phasic responses in primate neocortex. Exp. Brain Res. 114, 271–278. 10.1007/PL00005635 9166916

[B105] Schroeder C. E. Seto S. Arezzo J. C. Garraghty P. E. (1995). Electrophysiologic evidence for overlapping dominant and latent inputs to somatosensory cortex in squirrel monkeys. J. Neurophysiol. 74, 722732. 10.1152/jn.1995.74.2.722 7472377

[B106] Schroeder C. E. Seto S. Garraghty P. E. (1997b). Emergence of radial nerve dominance in “median nerve cortex” after median nerve transection in an adult squirrel monkey. J. Neurophysiol. 77, 522526. 10.1152/jn.1997.77.1.522 9120595

[B107] Schwaber M. K. Garraghty P. E. Morel A. Kaas J. H. (1993). Neuroplasticity of the adult primate auditory cortex following cochlear hearing loss. Am. J. Otol. 14, 252258. 8372922

[B108] Shepherd J. D. (2012). Memory, plasticity and sleep - A role for calcium permeable AMPA receptors? Front. Mol. Neurosci. 5, 49. 10.3389/fnmol.2012.00049 22514518 PMC3324118

[B109] Takesian A. E. Kotak V. C. Sanes D.H. (2012). Age-dependent effect of hearing loss on cortical inhibitory synapse function. J. Neurophysiol. 107, 937–947. 10.1152/jn.00515.2011 22090457 PMC3289466

[B110] Tanaka H. Grooms S. Y. Bennett M. V. Zukin R. S. (2000). The AMPAR subunit GluR2: still front and center-stage. Brain Res. 886, 190–207. 10.1016/S0006-8993(00)02951-6 11119696

[B111] Tocco G. Maren S. Shors T. J. Baudry M. Thompson R. F. (1992). Long-term potentiation is associated with increased [3H]AMPA binding in rat hippocampus. Brain Res. 573, 228–234. 10.1016/0006-8993(92)90767-4 1380390

[B112] Wall J. T. Felleman D. J. Kaas J. H. (1983). Recovery of normal topography in the somatosensory cortex of monkeys after nerve crush and regeneration. Science 221, 771–773. 10.1126/science.6879175 6879175

[B113] Wall J. T. Huerta M. F. Kaas J. H. (1992a). Changes in the cortical map of the hand following postnatal median nerve injury in monkeys: modification of somatotopic aggregates. J. Neurosci. 12, 3445–3455. 10.1523/JNEUROSCI.12-09-03445.1992 1527589 PMC6575718

[B114] Wall J. T. Huerta M. F. Kaas J. H. (1992b). Changes in the cortical map of the hand following postnatal ulnar and radial nerve injury in monkeys: organization and modification of nerve dominance aggregates. J Neurosci. 12:3456–3465. 1527590 10.1523/JNEUROSCI.12-09-03456.1992 PMC6575727

[B115] Wall P. D. (1977). The presence of ineffective synapses and the circumstances which unmask them. Philos. Trans. B 278, 361–372. 10.1098/rstb.1977.0048 19789

[B116] Wall P. D. Egger M. D. (1971). Formation of new connections in adult rat brain after partial deafferentation. Nature 232, 542–545. 10.1038/232542a0 4328622

[B117] Warren R. A. Dykes R. W. (1992). Population analysis of single neurons in cat somatosensory cortex. Somatosens. Motor Res. 9, 297–312. 10.3109/08990229209144779 1362827

[B118] Wellman C. L. Arnold L. L. Garman E. E. Garraghty P. E. (2002). Acute reductions in GABAA receptor binding in layer IV of adult primate somatosensory cortex after peripheral nerve injury. Brain Res. 954, 68–72. 10.1016/S0006-8993(02)03343-7 12393234

[B119] Whitney N. P. Peng H. Erdmann N. B. Tian C. Monaghan D. T. Zheng J. C. (2008). Calcium-permeable AMPA receptors containing Q/R-unedited GluR2 direct human neural progenitor cell differentiation to neurons. FASEB J. 22, 2888–2900. 10.1096/fj.07-104661 18403631 PMC2493446

[B120] Wierenga C. J. Ibata K Turrigiano G. G. (2005). Postsynaptic expression of homeostatic plasticity at neocortical synapses. J. Neurosci. 25, 2895–2905. 10.1523/JNEUROSCI.5217-04.2005 15772349 PMC6725152

[B121] Wiesel T. N. Hubel D. H. (1963a). Effects of visual deprivation on morphology and physiology of cells in the cat's lateral geniculate body. J. Neurophysiol. 26, 978–993. 10.1152/jn.1963.26.6.978 14084170

[B122] Wiesel T. N. Hubel D. H. (1963b). Single-cell responses in striate cortex of kittens deprived of vision in one eye. J. Neurophysiol. 26, 1003–1017. 10.1152/jn.1963.26.6.1003 14084161

[B123] Wong-Riley M. T. Jacobs P. (2002). AMPA glutamate receptor subunit 2 in normal and visually deprived macaque visual cortex. Vis. Neurosci. 19, 563–573. 10.1017/S0952523802195022 12507323

[B124] Zhou X. Panizzutti R. de Villers-Sidani E. Madeira C. Merzenich M. M. (2011). Natural restoration of critical period plasticity in the juvenile and adult primary auditory cortex. Neuroscience 31, 5625–5634. 10.1523/JNEUROSCI.6470-10.2011 21490203 PMC3758576

